# RNase Y Autoregulates Its Synthesis in *Bacillus subtilis*

**DOI:** 10.3390/microorganisms11061374

**Published:** 2023-05-24

**Authors:** Anna Korobeinikova, Soumaya Laalami, Clément Berthy, Harald Putzer

**Affiliations:** 1 Expression Génétique Microbienne, Institut de Biologie Physico-Chimique, CNRS, Université Paris Cité, 75005 Paris, France; anny.dudnic@gmail.com (A.K.); laalami@ibpc.fr (S.L.); berthy.clement@gmx.fr (C.B.); 2 Inovarion, 75005 Paris, France

**Keywords:** RNase Y, mRNA degradation, bacillus subtilis, ribonuclease

## Abstract

The instability of messenger RNA is crucial to the control of gene expression. In *Bacillus subtilis*, RNase Y is the major decay-initiating endoribonuclease. Here, we show how this key enzyme regulates its own synthesis by modulating the longevity of its mRNA. Autoregulation is achieved through cleavages in two regions of the *rny* (RNase Y) transcript: (i) within the first ~100 nucleotides of the open reading frame, immediately inactivating the mRNA for further rounds of translation; (ii) cleavages in the *rny* 5′ UTR, primarily within the 5′-terminal 50 nucleotides, creating entry sites for the 5′ exonuclease J1 whose progression is blocked around position −15 of the *rny* mRNA, potentially by initiating ribosomes. This links the functional inactivation of the transcript by RNase J1 to translation efficiency, depending on the ribosome occupancy at the translation initiation site. By these mechanisms, RNase Y can initiate degradation of its own mRNA when the enzyme is not occupied with degradation of other RNAs and thus prevent its overexpression beyond the needs of RNA metabolism.

## 1. Introduction

Control of gene expression requires the instability of messenger RNA. In bacteria, mRNA half-lives vary from seconds to over an hour. The decay of mRNA generally follows an “all-or-none” pattern, implying that if control is to be efficient, it must occur at the rate-limiting initial step of the degradation process. Studies in *Escherichia coli* and *Bacillus subtilis*, two evolutionary very distant organisms, have provided essential knowledge on the initiation of mRNA decay by endonucleolytic cleavage that can be summarized by “different enzymes—similar strategies”. The crucial role of the endoribonucleases RNase E (*E. coli*) and RNase Y (*B. subtilis*) in producing short-lived decay intermediates is now clearly recognized [1]. In *B. subtilis*, RNase Y affects global mRNA stability by cleaving its substrates with an RNase E-like single strand-specific cleavage specificity [2]. The intracellular levels of a majority of transcripts are affected in RNase Y-depleted strains [3,4,5]. An alternative degradation pathway has been described for some mRNAs where the decay of the primary transcript is triggered by the RNA pyrophosphohydrolase RppH which generates a monophosphorylated intermediate that becomes vulnerable to 5′-exonucleolytic digestion by RNase J1 [6]. RNase J, comprising RNase J1 and J2 subunits, is a multifunction enzyme with both endonuclease and 5′ exonuclease activity; the latter activity being essentially a property of the J1 subunit that requires a monophosphorylated 5′-end (or 5′-OH) [7,8,9,10]. RNase J1 thus functions primarily in the 5′ exonucleolytic degradation of RNA decay intermediates generated by endonucleolytic cleavage or 5′-end conversion. Interestingly, a monophosphorylated RNA 5′ end also potentiates the endonucleolytic activity of RNase Y in vitro [2], but it is unknown whether this plays a significant role in the RNase Y-dependent degradation of RNAs in vivo.

The sequence specificity of RNase Y in *B. subtilis* and other Gram-positive organisms is rather loose with a preference for UA-rich single-stranded sequences often flanked by secondary structures [2,11,12,13,14]. The same description also holds for RNase E cleavage sites with a few differences in the proposed sequence context [15,16,17,18,19,20]. *E. coli* RNase E can effectively replace RNase Y in *B. subtilis*, enabling a surprising reversal of transcript profiles not only of individual genes but also on a genome-wide scale [21].

Other important features shared between RNase Y and RNase E include a pseudo-compartmentalization; both enzymes are tethered to the inner cell membrane [22,23], in the case of RNase Y, via a single membrane-traversing N-terminal helix [24], (RNase Y is called YmdA in this reference). This is coherent with the predominant distribution of translating ribosomes around the cell periphery [25,26]. RNase Y diffuses rapidly across the membrane in the form of dynamic short-lived foci that become more abundant and increase in size following transcription arrest [27]. A degradosome complex centered on RNase Y has been proposed [28,29] but, unlike the RNase E-based degradosome in *E. coli* [30,31], cannot be isolated in the absence of crosslinking agents. Whether RNase Y can interact with other ribonucleases in any meaningful way remains an open question [32,33,34]. However, three small proteins, YlbF, YmcA, and YaaT forming the so-called Y-complex [35,36] were shown to alter RNase Y activity in vivo [37] by affecting the abundance of certain riboswitches and favoring the efficient maturation of operon mRNAs [13]. These effects of the Y-complex might be achieved by shifting the assembly status of RNase Y towards fewer and smaller membrane tethered complexes [21].

As the key ribonuclease initiating mRNA decay in *B. subtilis*, there is a need to keep the activity of RNase Y in a tightly controlled steady supply. Here, we show that RNase Y can cleave its own mRNA at several positions, within the 5′UTR and the open reading frame. This homeostatic mechanism allows tight regulation of its synthesis by modulating the decay rate of the *rny* (RNase Y) mRNA in response to changes in cellular RNase Y activity.

## 2. Materials and Methods

### 2.1. Bacterial Strains and Growth Conditions

The *B. subtilis* strains used in this work are derivatives of strain SSB1002, a wild-type laboratory stock strain derived from strain 168. *E. coli* strains JM109 [38] and XL1-Blue (Stratagene) were used for plasmid constructions and site-directed mutagenesis experiments. *E. coli* recA^+^ strain JM101 [39] was used for plasmid purification before transformation into *B. subtilis* cells. *B. subtilis* and *E. coli* strains were grown at 37 °C in LB medium with aeration to mid-log phase (OD_600_~0.5–0.6). Strains carrying the replicative control plasmid pWH353m (pC) or its *rny*-expressing pHMD22 (pY) or pHMD33 (pY*) derivative plasmids were grown without or with inducer (0.2 μg/mL of tetracycline). Antibiotics were added, at the following concentrations: ampicillin (100 µg/mL), kanamycin (5 µg/mL), chloramphenicol (5 µg/mL), neomycin (7 µg/mL), and spectinomycin (100 μg/mL) for *B. subtilis*, and ampicillin (200 μg/mL) and kanamycin (100 μg/mL) for *E. coli*.

Translational *rny-lac*Z fusions (see plasmids) are based on plasmid pHM14 [40] and were integrated in single copy at the *amyE* locus of the *B. subtilis* SSB1002 strain (see Table 1). Insertions were confirmed by PCR and sequencing. For the long *rny-lac*Z fusion and its mutated variants, the products of the ligation reaction were amplified with primers HP2248 and HP2249 and used to transform SSB1002 directly. Primers used in this study are listed in Table 2.

A *rny* deletion (∆*rny*::*spc*) strain was constructed by transforming *B. subtilis* wild-type and derivative strains with a DNA cassette composed of the spectinomycin resistance gene (*spc*) flanked by ~1 kb sequences corresponding to the up- and downstream regions of the *rny* ORF on the chromosome. The final fragment was obtained by overlapping PCR of three individual fragments. The 5′ and 3′ flanking regions were amplified using primer pairs HP2078-HP2080 and HP2104Rev-HP2083, respectively, the *spc* cassette was amplified from plasmid pDG1727 [41] with primers HP2079-HP2104. A fragment composed of the *spc* cassette and the 3′ region of *rny* was amplified with primers HP2079-HP2083 using the individual overlapping fragments. Finally, the full-length DNA cassette was amplified with oligonucleotides HP2078-HP2083 and two corresponding fragments from the first and second step as the DNA template.

The *rnjA* deletion strain SSB1086 (∆*rnjA::tet*) was constructed by transforming strain SSB525 with plasmid pHMJ26 which replaces the *rnjA* ORF with the tetracycline resistance cassette by double cross-over recombination.

### 2.2. Plasmid Constructs

*pHMD17.* Plasmid pHMD17 contains the entire *rny* promoter region with the *rny* ORF up to codon 5 inserted as a BamHI-EcoRI fragment (primers HP1714-HP1715) in-frame into the *lacZ* fusion vector pHM14 [40].

*pHMD22*. Plasmid pHMD22 contains the *rny* gene including the Shine–Dalgarno sequence as a 1.6 kb PCR fragment (primers HP1832-HP1833) inserted into the inducible expression vector pWH353m between sites SphI and SalI.

*pHMD28*. Plasmid pHMD28 contains the entire *rny* promoter region with the *rny* ORF up to codon 13 inserted as a BamHI-EcoRI fragment (primers HP2164-HP2165) in-frame into the *lacZ* fusion vector pHM14 [40].

*pHMD29*. Plasmid pHMD29 contains the entire *rny* promoter region with the *rny* ORF up to codon 31 inserted as a BamHI-EcoRI fragment (primers HP1714-HP2166) in-frame into the *lacZ* fusion vector pHM14 [40].

*pHMD33.* Plasmid pHMD33 is similar to pHMD22 but contains a mutated *rny* gene which codes for an inactive RNase Y (mutations H368A and D369A in HD domain). Two overlapping PCR fragments (primer pairs HP1832-HP2228 and HP2227-HP1833) containing the *rny* double mutations at the overlap were amplified from chromosomal DNA using KOD DNA polymerase (Novagen). Both fragments were used as the template for amplification with primers HP1832-HP1833. The resulting fragment was digested with SalI and SphI and ligated into the respective sites of plasmid pWH353.

*pHMD37.* Plasmid pHMD37 contains the entire *rny* promoter region with the *rny* ORF up to codon 210 inserted as a BamHI-EcoRI fragment (primers HP1714-HP1801) in-frame into the *lacZ* fusion vector pHM14 [40]. The ligation mix was amplified with primers HP2248-HP2249 and the resulting fragment used for transformation.

*pHMD38.* Plasmid pHMD38 is similar to pHMD37 but lacks a large part of the *rny* 5′ UTR (deletion between positions −20 and −158). A PCR fragment (primers HP1714-HP2269) was amplified and used as a megaprimer in a second PCR reaction with primer HP1801 using plasmid pHMD37 as the template. The resulting fragment was inserted as a BamHI-EcoRI fragment in-frame into the *lacZ* fusion vector pHM14 [40].

*pHMD39*. Plasmid pHMD38 is similar to pHMD17 but lacks a large part of the *rny* 5′ UTR (deletion between positions −20 and −158). A PCR fragment (primers HP1714-HP2268) was amplified plasmid pHMD17 as the template and inserted as a BamHI-EcoRI fragment in-frame into the *lacZ* fusion vector pHM14 [40].

*pHMJ26.* Plasmid pHMJ26 contains 3 fragments in the following order cloned into the vector pJRD184 [42] between the sites EcoRI and XbaI: a 0.7 kb EcoRI-PstI PCR fragment (primers HP899-HP1024) containing sequences upstream of *rnjA*, a 2.1 kb PstI-XhoI fragment of plasmid pDG1515 [41] containing the tetracycline resistance cassette and a 0.7 kb XhoI-XbaI PCR fragment (primers HP1039-HP1040) containing sequences downstream of *rnjA.*

*pWH353m.* Plasmid pWH353m is a modified version of the pWH353 vector [43] where the *cat* cassette was replaced between sites SmaI-StuI with the *mls* resistance cassette (sites StuI-SmaI) of plasmid pDG1663 [44].

### 2.3. Western Blot

For Western blot analysis, 20 μg of protein extract prepared as described previously [45] was separated by SDS-PAGE (10%). After electrophoretic transfer of the proteins, the nitrocellulose membrane (GE Healthcare, Chicago, IL, USA) was stained with amido black to check for equal transfer across all lanes. The membrane was blocked for 1 h with 5% skimmed milk in phosphate-buffered saline (PBS)-Tween buffer (100 mM NaH_2_PO_4_-Na_2_HPO_4_, pH 7.4, 100 mM NaCl, 0.1% Tween) and incubated with a monoclonal RNase Y antibody diluted in PBS-Tween for at least 4 h. Signals were detected by ECL Chemiluminescence (BIO-RAD, Clarity^TM^ Western ECL) associated with a CCD camera (BIO-RAD ChemiDoc XR System+). When necessary, ECL-detected proteins were quantified using the ImageLab software version 6.0.1 (Bio-Rad, Hercules, CA, USA). The uncropped versions of the Western blots are shown in Supplementary Materials.

### 2.4. Northern Blot

RNA blot analysis was carried out using 5–10 μg of total RNA isolated as described previously [40], separated on a 1% agarose/0.22 M formaldehyde gel, and blotted onto Hybond N+ membranes (GE Healthcare). RNA was crosslinked to the membrane at 120 mJ cm^–2^ for 1 min. RNA probes were denatured at 80 °C for 3 min, kept on ice for 2 min, and then added to the prehybridized membranes (Amersham Rapid-hyb buffer) for hybridization at 70 °C for 2 h. Membranes were washed at 68 °C with washing solutions I (2 × SSC and 0.1% SDS), II (1 × SSC, 0.1% SDS), and III (0.5 × SSC, 0.1% SDS) for 15 min each. The signals were detected with a GE Healthcare Typhoon FLA 9500 imaging system. As lacZ and 5′ UTR *rny* probes, T7 RNA polymerase in vitro transcripts were used which were generated from a PCR template obtained with oligonucleotides HP2270-HP2271 and HP2238-HP2239, respectively. Chromosomal DNA of the *B. subtilis* SSB530 strain was used as a template for PCR. 16S rRNA was used as loading control (hybridization with oligonucleotide HP2113). The uncropped versions of the Northern blots are shown in Supplementary Materials.

### 2.5. Half-Life Measurements

For half-life measurements, rifampicin (Sigma, St. Louis, MO, USA) was added at 150 µg/mL to cultures at OD_600_ ~0.5. At 0, 1, 2, 4, 6, and 10 min after addition of rifampicin, 10 mL of cell culture were mixed with 5 mL of frozen buffer (20 mM Tris-HCl pH 7.8, 5 mM MgCl_2_, 20 mM NaN_3_), centrifuged, and frozen in dry ice/ethanol. Total RNA from the samples was purified and analyzed by Northern blot as described above.

### 2.6. Primer Extension Analysis

Primer extension was carried out using 5 μg of total RNA, 5′[^32^P]-labelled primer HP905 complementary to sequences at the 5′ end of the *lacZ* ORF and Superscript III reverse transcriptase (Invitrogen, Waltham, MA, USA) following the instructions of the manufacturer. Reactions were carried out at 55 °C for 10 min.

### 2.7. β-Galactosidase Measurements

For β-galactosidase measurements, cells were grown in the appropriate medium and cell extracts were prepared from 3 to 5 mL of cell culture. Extracts were used for measurements as described elsewhere [46]. All reported values are based on two to four independent measurements. Error bars represent standard deviation (sd).

## 3. Results

### 3.1. Rny-lacZ Expression Is Responsive to RNase Y Levels In Vivo

**Figure 1:**
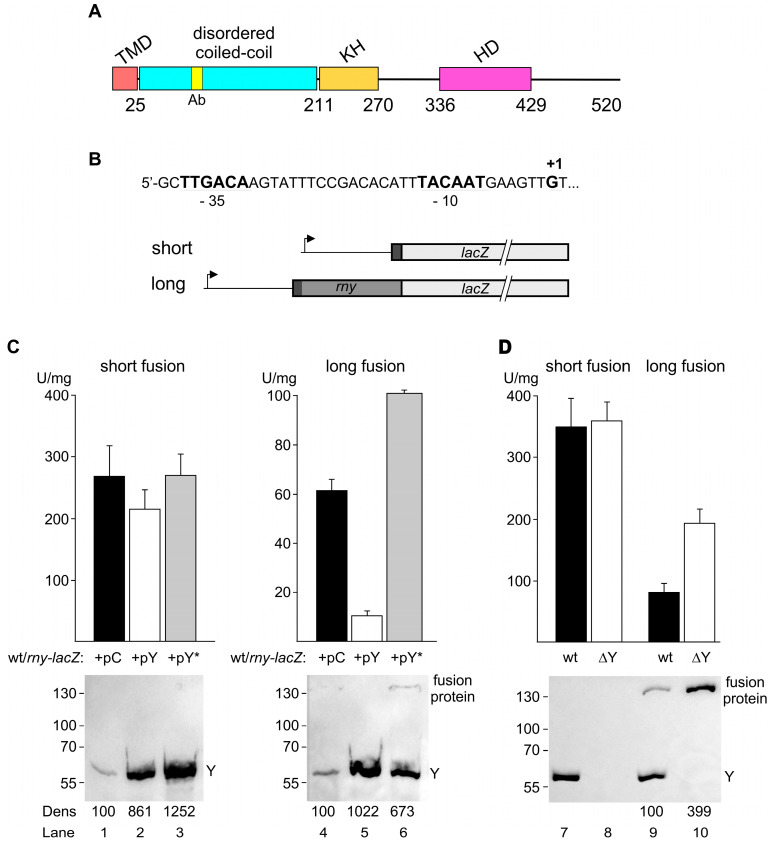
Effect of RNase Y on the activity of *rny-lac*Z fusions. (**A**). Domains composing *B. subtilis* RNase Y (520 aa) include an N-terminal transmembrane domain (TMD, aa 1–25), followed by a large region predicted to be disordered (aa~30–210), an RNA binding KH domain (aa 211–270), and a metal-chelating HD domain (aa 336–429) containing the conserved His/Asp motif required for RNase activity [2,24,48,49,50]. Ab indicates the position of the 12 aa peptide used for monoclonal antibody production. (**B**). Sequence of the sigma A-type promoter of the *rny* gene and schematic of the «short» and «long» translational *rny-lacZ* fusions; the −10 and −35 regions and the transcription start site (+1) are indicated. (**C**). β-galactosidase activity of short and long *rny-lac*Z fusions in strains containing either the empty control plasmid (pC) or plasmids expressing wild-type RNase Y (pY) or an inactive version of RNase Y (pY*) in the presence of inducer (0.2 mg/mL of tetracycline). All cultures were grown in the presence of inducer and the amount of RNase Y in the cell was determined by Western blot using an RNase Y-specific antibody (lower panels). The antibody also detected the long *lacZ* fusion protein. (**D**). β-galactosidase activity of long and short *rny-lac*Z fusions in the wild-type and *rny* deletion (∆Y) strain. RNase Y expression and the absence of RNase Y in the mutant strain was verified by Western blot (lower panel). Y = RNase Y. Numbers below the Western blots show relative densitometry readings (Dens) based on the control value arbitrarily set to 100 in each gel. Measurements refer to the bands corresponding to RNase Y, except in (**D**) where they correspond to the fusion protein.

We first assessed the ability of RNase Y to regulate its own expression using two translational *lacZ* fusions. Both contained the entire 164 nt 5′ UTR of the *rny* gene identified previously by a transcriptomic analysis [47] and confirmed by primer extension analysis (see below). Transcription is driven by a typical sigma A-type promoter (Figure 1B). The two *lacZ* constructs differed in the length of the *rny* ORF fused in-frame to *lacZ*, i.e., 5 and 210 codons, referred to as short and long fusions, respectively (Figure 1B). The fusions were integrated in single copy at the *amyE* locus.

Regulated overexpression of RNase Y was achieved by further transforming *rny-lacZ*-containing strains with the *rny* expression plasmid pHMD22 that allows transcriptional control of the additional plasmid-borne *rny* gene by a tetracycline-inducible promoter. Growth in the presence of inducer increased the intracellular RNase Y level up to about 8- to 10-fold (Figure 1C, lower panels, confer lanes 1, 2 and 4, 5). This overexpression of RNase Y only slightly reduced the activity of the short *rny-lacZ* fusion (~1.3-fold) while the long *rny-lacZ* fusion was strongly (~6-fold) repressed (Figure 1C, β-gal activities at the top).

To test whether this control requires the catalytic activity of RNase Y we also overproduced, an RNase Y protein where the conserved HD motif in the active site was mutated (H368A/D369A, Figure 1A). We have previously shown that single mutations of the HD motif almost abolish the endonucleolytic activity of RNase Y [2]. In contrast to the wild-type enzyme, overexpressing the inactive form of RNase Y (Figure 1C, lanes 3 and 6) does not decrease the activity of the short nor the long form of the *rny-lacZ* fusions. On the contrary, expression of the long *lacZ* fusion was significantly increased under these conditions, suggesting that inactive RNase Y interferes with the activity of the chromosome-encoded wild-type form of the enzyme (Figure 1C, see also Discussion). This increase in β-galactosidase activity is also visible in the Western blot analysis aimed at quantifying RNase Y overproduction (Figure 1C, Western blots at the bottom, cf. lanes 5 and 6). Since the monoclonal antibody is directed against a peptide in the N-proximal 210 aa (but not within the N-terminal 5 aa) of RNase Y (Figure 1A), we could also detect the fusion protein synthesized from the long but not the short form of the *rny-lacZ* fusion construct (Figure 1C, lower panel, lane 6; see also lower WB panel of Figure 1D).

We next analyzed the effect of the absence of RNase Y on *rny-lacZ* expression. For this purpose, the entire *rny* ORF was replaced with a spectinomycin resistance cassette. Expression of the short *rny-lacZ* fusion remained unchanged in the absence of RNase Y. However, expression of the long fusion was induced 2- to 3-fold when measuring both β-galactosidase activity and the level of fusion protein in the cell by Western blot, respectively (Figure 1D, lanes 9 and 10).

These data indicated that RNase Y might autoregulate its own expression and that control primarily involves *rny* mRNA sequences between codon 5 and 210.

### 3.2. RNase Y Affects rny and rny-lacZ Transcript Levels Alike

**Figure 2:**
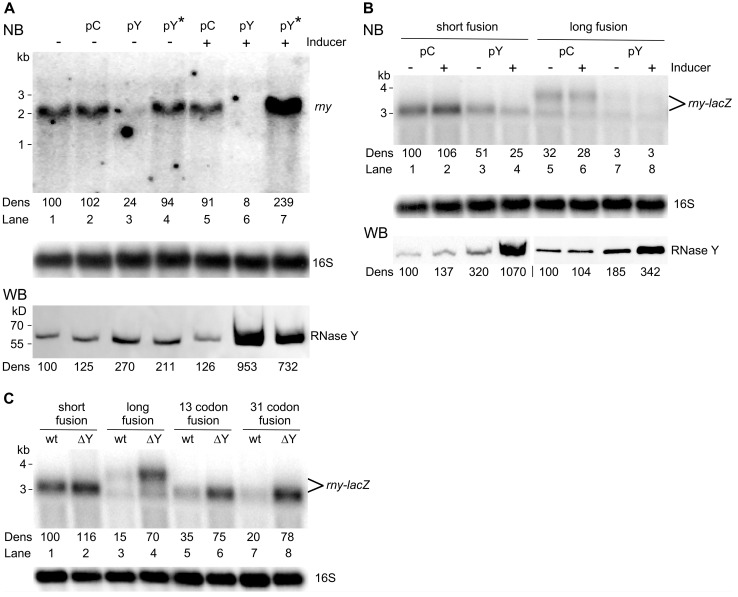
Effect of RNase Y on wild-type *rny* and *rny-lacZ* transcript levels. (**A**). Northern blot analysis of *rny* mRNA (1.72 kb) in the wild-type (SSB1002) and strains containing either the control plasmid (pC, SSB614) or plasmids conditionally overexpressing wild-type RNase Y (pY, SSB615) or an inactive version of RNase Y (pY*, SSB616). Cultures were grown with or without inducer (0.2 μg/mL tetracycline) as indicated. RNase Y levels were determined by Western blot (lower panel) (**B**). Northern blot analysis of short and long *rny-lac*Z transcripts (3.2 and 3.8 kb, respectively) in strains containing either the control (pC, SSB1082 and SSB1084) or the RNase Y conditionally overexpressing plasmid (pY, SSB1083 and SSB1085). Transcripts were detected using a *lacZ-*specific probe. Cultures were grown with or without inducer (0.2 μg/mL tetracycline) as indicated. RNase Y levels were determined by Western blot (lower panel) (**C**). Northern blot analysis of *rny-lac*Z transcripts in wild-type and a Δ*rny* background using a *lacZ-*specific probe. Wild-type strains (lanes 1, 3, 5, 7): SSB525, SSB530, SSB599, SSB600. Delta-rny strains (lanes 2, 4, 6, 8): SSB593, SSB594, SSB602, SSB603. All cultures were grown in LB medium. Hybridization with a 16S rRNA specific probe was used as a loading control. Numbers below the Northern and Western blots show relative densitometry readings based on the control value arbitrarily set to 100 in each gel. Measurements for Northern blots are already corrected for the 16S loading controls. NB: Northern blot. WB: Western blot.

The data obtained with the short *rny-lacZ* fusion described above strongly suggested that the *lacZ* mRNA part of the fusion mRNA does not contribute to autoregulation (see also discussion). Nevertheless, to test how RNase Y affects its own expression, we analyzed the effect of RNase Y on both *rny* and *rny-lacZ* mRNA levels. First, we transformed the wild-type strain (SSB1002) with plasmid pHMD22 for inducible RNase Y overexpression (pY) or with the empty vector as control (pC). The *rny* messenger transcribed from the chromosomal gene was detected with a probe specific for the *rny* 5′UTR which does not detect *rny* mRNA transcribed from the plasmid since this gene copy lacked the 5′ UTR. The *rny* mRNA level transcribed from the chromosome was very sensitive to the intracellular RNase Y concentration (Figure 2A). In the absence of inducer, we observed a two- to three-fold overproduction of RNase Y due to leaky expression. This was already sufficient to strongly reduce chromosomal *rny* mRNA about four-fold (Figure 2A). In the presence of inducer RNase Y was overproduced ~ten-fold, causing *rny* mRNA to drop below detectable levels (Figure 2A). In contrast, a similar overproduction of the inactive RNase Y mutant led to a more than two-fold increase in *rny* transcripts (Figure 2A), mirroring a similar effect on the β-galactosidase expression of the long *rny-lacZ* fusion under the same conditions (Figure 1C). The transcripts of the short and long *rny-lacZ* fusions, detected using a *lacZ*-specific probe, responded in much the same way as the *rny* mRNA to overproduction of RNase Y (Figure 2B).

While the long fusion mRNA became almost undetectable when overproducing RNase Y, the short fusion mRNA was also reduced two-fold suggesting that RNase Y does not only cleave within the *rny* ORF but also in the 5′ UTR (Figure 2B). In the absence of RNase Y (Figure 2C), the short *rny-lacZ* fusion mRNA is barely increased (~1.1-fold) while induction of the long *rny-lacZ* fusion mRNA is upregulated by a factor of 5-fold compared to a wild-type strain, again suggesting that 5′ proximal sequences in the *rny* ORF play an important role for controlling the *rny* transcript levels in the cell. The similar response of *rny* mRNA and the long *rny-lac*Z transcript to changes in the cellular level of RNase Y indicated that the regulation of the long *rny-lac*Z fusion by RNase Y quite accurately mimics autoregulation of the wild-type *rny* gene. Intermediate length *rny-lac*Z fusions containing 13 and 31 *rny* codons, respectively, showed that with increasing length regulation becomes more efficient. The 31 codon fusion was regulated to the same extent as the long fusion suggesting that cleavages within the first 100 nucleotides of the *rny* ORF are important for control.

### 3.3. RNase Y Affects the Stability of Its Own mRNA

**Figure 3:**
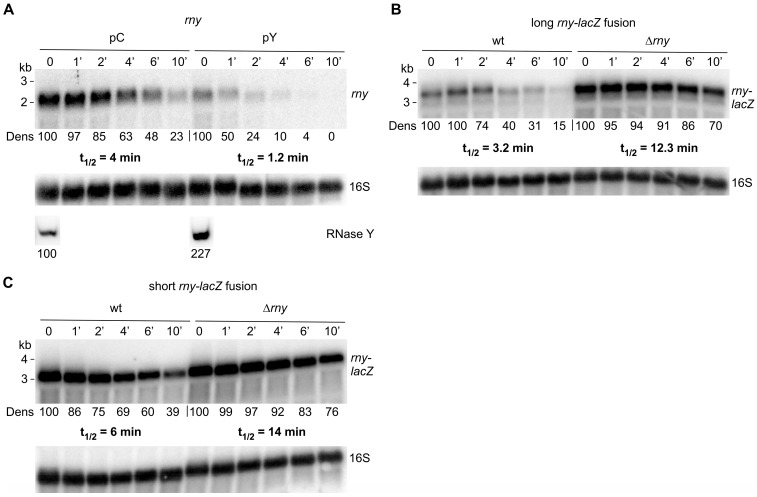
Effect of RNase Y levels on the half-life of *rny* and *rny-lacZ* transcripts. (**A**). Half-life of wild-type *rny* mRNA in *B. subtilis* wild-type strains harboring either the empty vector (pC, SSB614) or the RNase Y overproducing plasmid (pY, SSB615). Rifampicin was added to mid-log cultures (time 0′) and RNA isolated at the indicated time points was hybridized to a probe specific for the *rny* 5′ UTR; this probe detects the chromosomally encoded *rny* mRNA and the *rny-lacZ* fusion mRNAs, but not the plasmid-encoded *rny* mRNA. The intracellular RNase Y level at time 0 was determined by Western blot (lower panels). The position of the *rny* transcript on the Northern blots is indicated. (**B**). Half-life measurement of the long *rny-lacZ* fusion mRNA in wild-type (SSB530) and ∆*rny* (SSB594) strains. Total RNA was isolated at the indicated time points and *rny-lacZ* transcripts were detected with a *lacZ-*specific probe. (**C**). Half-life measurement of the short *rny-lacZ* fusion mRNA in wild-type (SSB525) and ∆*rny* (SSB593) strains. Transcripts were detected with a *lacZ-*specific probe. All cultures were grown in LB medium and no inducer was added for RNase Y overexpression. Hybridization with a 16S rRNA specific probe was used as a loading control. Numbers below the Northern and Western blots (Dens) show relative densitometry readings based on the control value arbitrarily set to 100 in each gel. Measurements for Northern blots are already corrected for the 16S loading controls.

As shown above, it is the endonucleolytic activity of RNase Y which is required for the observed auto-control of *rny* expression. We therefore investigated whether changes in *rny* transcript levels involve modulation of *rny* mRNA stability. The half-life of the chromosomal *rny* transcript was measured in presence of physiological levels of RNase Y, i.e., *B. subtilis* cells containing the empty control plasmid (pC), or when the nuclease was overproduced (pY). Overproduction of RNase Y from pY is induced with tetracycline but since the high amount of the nuclease expressed under these conditions lead to undetectable *rny* mRNA levels (confer Figure 2A) we grew cells without inducer. As already mentioned, this still leads to ~two-fold excess of RNase Y in the cell. This overproduction of RNase Y caused a 3.5-fold decrease in *rny* half-life (1.2 min vs. 4 min) as shown in Figure 3A. We observed an equivalent four-fold effect when measuring the half-life of the long *rny-lacZ* fusion (Figure 3B), but a smaller two-fold reduction in half-life for the short *rny-lacZ* fusion (Figure 3C). This suggested that cleavage(s) in the 5′ proximal sequences of the *rny* ORF make the most significant contribution to auto-control.

### 3.4. RNase Y Can Cleave Efficiently in the 5′ UTR of its mRNA

**Figure 4:**
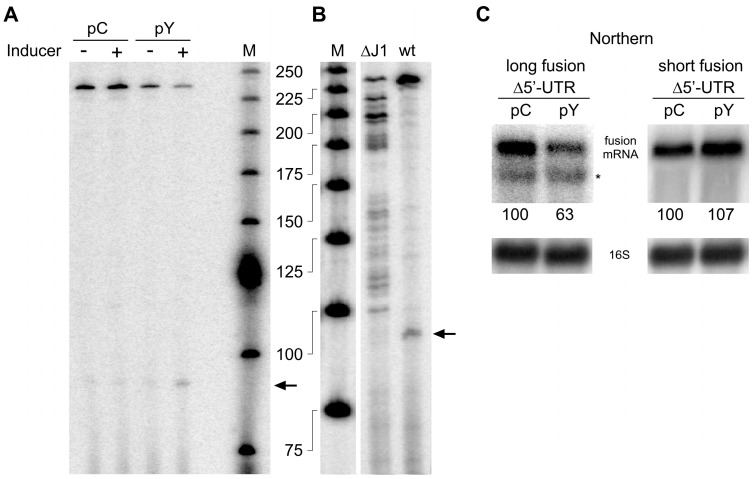
RNase Y cleaves at multiple sites in the *rny* 5′ UTR. (**A**). Primer extension analysis of the short *rny-lacZ* fusion mRNA using a *lacZ-*specific primer (HP905, the 5′ end of the primer is complementary to *lacZ* sequences 57 nt downstream of the last *rny* codon) in strains harboring the empty control plasmid (pC, SSB1082) or the conditionally RNase Y overexpressing plasmid (pY, SSB1083). Cells were grown in the absence or presence of inducer (0.2 mg/mL of tetracycline). An arrow indicates the putative mRNA 5′ end generated by the 5′ exonuclease activity of RNase J1 and arrested by a ribosome bound to the Shine–Dalgarno sequence (15 nucleotides upstream of the AUG initiation codon corresponds to a cDNA of 88 nt). (**B**). Primer extension analysis of the short *rny-lacZ* fusion mRNA using a *lacZ-*specific primer (HP905) in a wild-type (SSB525) and RNase J1 mutant strain (∆J1, SSB1086). Extension of the primer to the original 5′ end leads to a product of 238 nt. The major 5′ end within the 5′ UTR likely generated by 5′ exonuclease trimming is indicated by an arrow. M: 25 bp ladder. (**C**). Effect of RNase Y overexpression on long and short *rny-lacZ* fusion transcripts that lack the *rny* 5′ UTR. Total RNA from strains carrying the long ∆5′ UTR *rny-lacZ* fusion and the empty control plasmid (pC, SSB638) or the RNase Y overexpression plasmid (pY, SSB639), and from strains carrying the short ∆5′ UTR *rny-lacZ* fusion and the empty control plasmid (pC, SSB630) or the RNase Y overexpression plasmid (pY, SSB631) was hybridized with a *lacZ*-specific probe. Numbers below the Northern blot show relative densitometry readings of the *lacZ* fusion mRNA based on the control value arbitrarily set to 100 in each gel. Measurements for Northern blots are already corrected for the 16S loading controls. Asterisks mark the position of ribosomal RNA hybridizing non-specifically to the *lacZ* probe.

The half-life and the intracellular level of the short *rny-lacZ* mRNA was still affected in the *∆rny* strain or by increased levels of RNase Y, albeit to a lesser extent than the long fusion mRNA. This implied that RNase Y might not only cleave within the *rny* open reading frame but also in the 5′ UTR. In order to identify such cleavages, we analyzed the short fusion mRNA by primer extension in strains containing the inducible RNase Y overexpression plasmid pHMD22 (pY) grown with or without inducer or the control plasmid. The amount of full-length cDNA (238 nt) was reduced in the RNase Y overexpressing strain, especially when grown in the presence of inducer (Figure 4A). Under these conditions, a ~90 nt cDNA accumulated as would be expected if RNase Y cleavage products were trimmed by RNase J1 to the point when the 5′ exonuclease encounters a ribosome bound to the translation initiation site. Since the ribosome protects about −15 to +15 nt on the mRNA with respect to the initiation codon [51], a 5′-end at position −15 would result in a cDNA of 88 nt which is in line with the detected ~90 nt cDNA product.

In order to find out where RNase Y might cleave within the 5′ UTR, we carried out the primer extension analysis in an RNase J1 deletion mutant where the 5′ ends of cleavage products should be stabilized. Surprisingly, we found a large number of cleavages all along the 164 nt 5′ UTR (Figure 4B). In accordance, the multiple new 5′ ends detected within the 5′ UTR are almost absent in the wild-type strain expressing RNase J1. Instead, the major 5′ end identified within the 5′ UTR of the *rny* mRNA likely results from trimming by RNase J1 up to the position of a ribosome bound at the Shine–Dalgarno sequence as described above (Figure 4B).

Deletion of almost the entire 5′ UTR, residues −21 to −158 of the 164 nt leader, in the long *rny-lacZ* construct still allowed auto-control when overexpressing RNase Y, albeit at some reduced level as judged by Northern analysis (Figure 4C, left panel). However, in the absence of the 5′ UTR the short *rny-lacZ* fusion mRNA level was completely insensitive to RNase Y overexpression (Figure 4C, right panel), as would be expected from the absence of both regions we had identified as being sensitive to cleavage by RNase Y.

## 4. Discussion

In this study, we have shown that *B. subtilis* RNase Y can regulate its expression by cleaving its own mRNA. The use of *rny-lacZ* translational fusions was helpful as notably the long fusion encoding the 210 N-terminal amino acids of RNase Y behaved very similar when compared to the wild-type *rny* transcript. Auto-control is very sensitive, a 1.5-fold overexpression of RNase Y is sufficient to strongly reduce *rny* mRNA levels and the same *rny* transcripts are strongly increased in a strain lacking RNase Y.

This regulation is most likely direct and strictly requires the endonucleolytic activity of the enzyme. Even a strong overexpression of a catalytic mutant of RNase Y does not downregulate *rny* expression. Interestingly, *rny* transcript levels or *rny-lacZ* fusion activity are not only not reduced under these conditions but strongly induced. This is exactly the phenotype we observed in a ∆*rny* strain and implies that inactive RNase Y has a dominant negative effect on global RNase Y activity. Soluble forms of RNase Y lacking the N-terminal transmembrane domain are active in vitro [2] and form mainly dimers and tetramers [49]. The N-terminal non-catalytic domain of RNase Y which is intrinsically disordered contributes significantly to dimerization [50]. In vivo, RNase Y also forms dynamic higher order complexes whose assembly status can be shifted by regulatory proteins, with smaller complexes likely being the most active form of the enzyme [27]. Since RNase Y occurs, at a minimum, as a dimer, overproduction of inactive RNase Y proteins should lead to mixed forms of RNase Y oligomers where the mutated entity might also inhibit the activity of the wild-type form present in the same complex. Alternatively, but not mutually exclusive, mutated RNase Y might simply compete with the wild-type enzyme for binding to the *rny* mRNA and other substrates, imitating an apparent lack of RNase Y in the cell.

The use of translational *rny-lacZ* fusions proved to be very useful for characterizing the regions of the *rny* transcript susceptible to RNase Y cleavage. Early studies in *B. subtilis* on the role of stabilizing elements like secondary structure or a ribosome binding site at the 5′ end of an mRNA had shown that the *E. coli lacZ* mRNA fused to these upstream elements could actually be stabilized to attain half-lives exceeding 40 min [52,53]. This suggested that in *B. subtilis* the heterologous *lacZ* mRNA is quite resistant to endonucleolytic cleavage, notably by RNase Y, an enzyme unknown at the time. Here, we could clearly show that even a strong overexpression of RNase Y cannot reduce the intracellular level of a *rny-lacZ* fusion mRNA when those *rny* regions that are susceptible to RNase Y cleavage are replaced or deleted (Figure 4C).

RNase Y can cleave its mRNA at multiple positions to control the steady-state level of the functional transcript. Cleavages within the open reading frame would inactivate the transcript for a new translational round. We tried to identify specific cleavage sites within the *rny* ORF by a primer extension analysis of *rny-lacZ* fusions of various length in an RNase J1 mutant strain and obtained multiple but not well reproducible signals. While the long fusion containing 600 nt of *rny* sequences was the most susceptible to RNase Y cleavage, a significant effect of the absence of RNase Y on the level of *rny-lacZ* fusion mRNA was already observed with a fusion containing only 39 nt (13 codons) of *rny*. The presence of 93 nt (31 codons) of *rny* gave results equivalent to those of the long fusion. This indicates that RNase Y controls its own expression to a large part by cleaving within the first 100 nucleotides of the *rny* ORF. This might involve multiple cleavages as the absence of a 5′ exonucleolytic activity (RNase J1 mutant) was not enough to precisely identify new 5′ ends within the ORF. However, internal fragments created by more than one cleavage would also be degraded by 3′ exoribonucleases complicating their detection. Translation of the *rny* ORF does not appear to play an important role in regulation. A long *lacZ* fusion construct with an AUC start codon mutation was downregulated when RNase Y was overexpressed, similarly to the wild-type fusion albeit at much lower expression levels.

A second more indirect control involves RNase Y cleavages within the *rny* 5′ UTR. The many new 5′ ends detected in the RNase J1 mutant may not all be genuine RNase Y cleavage sites. However, overexpression of RNase Y clearly destabilizes the 5′ UTR producing entry sites for the 5′ exonuclease J1 which trims the mRNA until encountering an initiating ribosome. It remains a surprising finding that a 3 kb mRNA molecule like the *lacZ* transcript appears completely resistant to cleavage by RNase Y while the 160 nt *rny* 5′ UTR is cleaved efficiently at multiple positions. RNase Y is thought to cleave with little sequence specificity in single-stranded AU-rich sequences with a secondary structure in the up- or downstream vicinity and with a certain preference for cleavage at or close to a G residue [2,11,12,14]. We have observed that the strongest cleavages in the *rny* 5′ UTR occur within the fifty 5′ proximal nucleotides, a region composed of 76% U and A residues with a single stem-loop in the middle part. The 5′ proximal sequences of the *rny* ORF also contain several long A stretches interrupted by a few G or C residues. However, as expected we found no clearly defined characteristics that would allow a valid prediction of potential RNase Y cleavage sites, or explain why the *lacZ* mRNA is not a substrate.

The β-gal activity of the *rny-lacZ* fusions was generally in line with the transcript levels determined by Northern blot except in one case. Induced overexpression of RNase Y caused only a minor 1.3-fold effect on the β-gal activity of the short fusion reporter while the same conditions led to a 4-fold reduction in the levels of the short fusion mRNA (Figure 2B, confer lanes 1 to 4). This apparent difference might be linked to the length of the *rny-lacZ* transcript (3.2 kb). Assuming an average elongation rate of ~40 nucleotides/s, it should take about 80 s to synthesize the 3.2 kb transcript. Notably under conditions of RNase Y overexpression, most nascent *rny-lacZ* transcripts should begin to decay before their synthesis is completed. With the short *rny-lacZ* fusion, RNase Y cannot directly inactivate the transcript because the *rny* sequences within the ORF susceptible to cleavage are absent. However, efficient cleavage within the *rny* 5′ UTR by RNase Y still occurs. In this case, inactivation of the transcript relies on the progression of the incoming 5′ exonuclease RNase J1 which can be hampered by a bound ribosome, notably at the Shine–Dalgarno sequence. In this configuration, the translation of most *rny-lacZ* mRNAs can probably proceed to the end abidingly, even though the levels of the full-length transcripts are constantly decreased due to shortening of the mRNA from the 5′ end. Since actively translating transcripts would be less efficiently degraded from the 5′ end this could explain the discrepancy between β-gal activity and full-length short *rny-lacZ* mRNA levels. In accordance, the 5′ dependent degradation is relieved in short *rny-lacZ* transcripts when the 5′ UTR *rny* sequences that are susceptible to RNase Y cleavage are not present.

**Figure 5:**
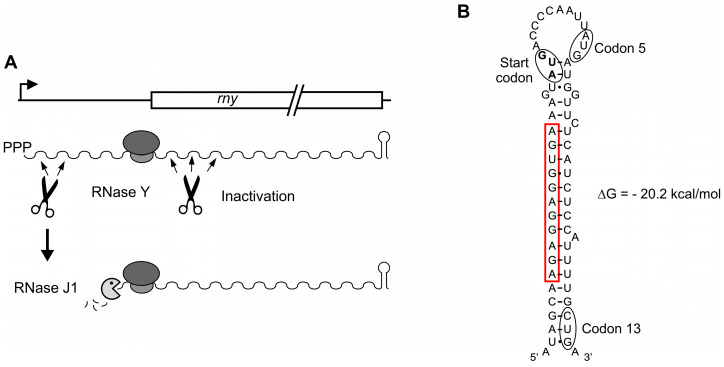
Model of autoregulation of RNase Y synthesis. (**A**). Freely available RNase Y can downregulate its own synthesis by cleaving within the ORF and the 5′ UTR. Cleavage in the ORF likely at multiple positions immediately inactivates the transcript. On the contrary, cleavage within the 5′ UTR generates 5′ monophosphorylated entry sites for the 5′ exoribonuclease activity of RNase J1. In this case, inactivation of the *rny* transcript depends on ribosome occupancy at the translation initiation site, hence linking the degradation of the *rny* mRNA to translation efficiency. (**B**). Potential secondary structure of the translation initiation region predicted by RNAfold [55]. The Shine–Dalgarno sequence is boxed in red.

We have summarized the current data in a model for the regulation of expression of the *rny* gene (Figure 5A). It contrasts with that proposed for the autoregulation of RNase E in *E. coli* where binding of a stem-loop in the *rne* mRNA 5′ UTR, without being cleaved, seems required for expediting cleavage elsewhere within the *rne* transcript [54].

In this context, it is noteworthy that the *rny* Shine–Dalgarno sequence can form 10 canonical Watson–Crick and one G•U base pair with the 3′-end of 16S rRNA, with a ∆G of −17.5 kcal mol^−1^. This actually represents one of the strongest ribosomal binding sites present in *B. subtilis* mRNAs [56]. A strong interaction between the ribosome and the Shine–Dalgarno sequence might be necessary for the ribosome to efficiently block RNase J1 progression, but also to provide for efficient translation initiation especially since the RBS can be part of a strong secondary structure that extends up to codon 13 of the *rny* ORF (Figure 5B). Similar structures are also found in the *rny* transcripts of closely related species. Indeed, the secondary structure itself may also decelerate RNase J1 progression and such a possibility would be compatible with the observed ~90 nt cDNA product from the primer extension experiment. On the other hand, formation of the stem-loop structure could also play a role for the susceptibility of the translated mRNA to RNase Y cleavages occurring in the 5′ proximal coding sequences. Mutations predicted to destabilize the structure without altering the RBS had no significant effect on expression but slightly reduced the capacity of overexpressed RNase Y to downregulate expression. The protection of the *rny* transcript from 5′ exonucleolytic degradation by ribosome binding provides a sensitive link between translation initiation and the functional stability of the RNA. A number of aspects of this regulation need to be addressed in the future, notably the role of secondary structure formation at the translation initiation site and whether other factors potentially interacting with RNase Y can interfere with autoregulation.

## Figures and Tables

**Table 1:** *B. subtilis* strains used in this study.

*B. subtilis* Strain	Relevant Genotype	Reference
SSB1002	Wild-type strain	Lab stock
SSB525	*amyE::rny-lacZ* (short, 5 aa)	This work
SSB530	*amyE::rny-lacZ* (long, 210 aa)	This work
SSB593	*amyE::rny-lacZ* (short, 5 aa), ∆*rny::spc*	This work
SSB594	*amyE::rny-lacZ* (long, 210 aa), ∆*rny::spc*	This work
SSB599	*amyE::rny-lacZ* (13 aa)	This work
SSB600	*amyE::rny-lacZ* (31 aa)	This work
SSB602	*amyE::rny-lacZ* (13 aa), ∆*rny::spc*	This work
SSB603	*amyE::rny-lacZ* (31 aa), ∆*rny::spc*	This work
SSB614	SSB1002, pWH353m	This work
SSB615	SSB1002, pHMD22	This work
SSB616	SSB1002, pHMD33	This work
SSB630	*amyE::rny-lacZ* (short, 5 aa, ∆5′ UTR), pWH353	This work
SSB631	*amyE::rny-lacZ* (short, 5 aa, ∆5′ UTR), pHMD22	This work
SSB638	*amyE::rny-lacZ* (long, 210 aa, ∆5′ UTR), pWH353	This work
SSB639	*amyE::rny-lacZ* (long, 210 aa, ∆5′ UTR), pHMD22	This work
SSB1082	*amyE::rny-lacZ* (short, 5 aa), pWH353m	This work
SSB1083	*amyE::rny-lacZ* (short, 5 aa), pHMD22	This work
SSB1084	*amyE::rny-lacZ* (long, 210 aa), pWH353m	This work
SSB1085	*amyE::rny-lacZ* (long, 210 aa), pHMD22	This work
SSB1086	*amyE::rny-lacZ* (short, 5 aa), ∆*rnjA::tet*	This work

“Short, 5aa” and “Long, 210 aa” refers to the number of *rny* codons present in the short and long *rny-lacZ* fusions, respectively.

**Table 2:** Oligonucleotides used in this study.

Oligonucleotide	Sequence 5′–3′
HP899	ACTGAGAATTCACGGTTTTTCTCGTACTTTCCGGT
HP905	GATTAAGTTGGGTAACGCCAGGGT
HP1024	ATTACCTGCAGACCGCTCATAACTCCAATAC
HP1039	ACTATCTCGAGTCCTGCCGATCATTATGGAGGT
HP1040	TAAGTTCTAGATGACGATGGTAAAAGGGCAGAC
HP1714	ATCGGAATTCTGATTGGCGATCTTCTTTTGG
HP1715	TATCGGATCCATAATTGGGGTCATACTTTCAC
HP1801	TATCGGATCCGTTGTTTCGGCAACGTGGTC
HP1832	ATCCGCATGCACCAAGTTCATAGCAAG
HP1833	ATACTGTCGACTAAACAAAAAACCCAGCTCATTAAGC
	TGGGTTTGCGCATCACTTTATTTTGCATACTCTACGGCT
	CGAGTC
HP2078	ACTGAGAATTCCGTAAACACTACATTCAAAATAATCC
HP2079	CATAGCAAGAGGAGGTGAAAGTATGAATACATACGAA
	CAAATTAATAAAG
HP2080	CTTTATTAATTTGTTCGTATGTATTCATACTTTCACCTCCT
	CTTGCTATG
HP2083	TAAGTTCTAGAGGATAAGTGAGTGTTCATTAGAAC
HP2104	AATTCCTTGCCGTCAAAAAAATAAAGTGATGCTTAGCGC
	ATCACTTTATAATTTTTTTAATCTGTTATTTAAATAGTTTA
	TAG
HP2104rev	CTATAAACTATTTAAATAACAGATTAAAAAAATTATAAA
	GTGATGCGCTAAGCATCACTTTATTTTTTTGACGGCAAGG
	AATT
HP2113	CAGCGTTCGTCCTGAGC
HP2164	ATCGGAATTCTGATTGGCGATCTTCTTTTGGATGAATTGC
HP2165	TATCGGATCCAGCAAAATGGAGATGAGAACCATCATAA
	TTGG
HP2227	GGGTCTTCTTGCCGCCATCGGGAAAGC
HP2228	GCTTTCCCGATGGCGGCAAGAAGACCC
HP2166	TATCGGATCCGCTTCGGCAATGGTTTTAC
HP2238	GTAACATTTGTTTTTTTAACATGATTG
HP2239	TTCAATTAATACGACTCACTATAGGTTGTTGCTTAAA
	AAGTGTC
HP2248	CTCCAGTCTTCACATCGGTTTG
HP2249	GGTTTTGGCCGACGCTGGATCTC
HP2268	TATCGGATCCATAATTGGGGTCATACTTTCACCTCCTCTT GCTATGTTACAACTTCATTGTAAATGTGTCGG
HP2269	GGGTCATACTTTCACCTCCTCTTGCTATGTTACAACTTCA
	TTGTAAATGTGTCGGAAATACTTG
HP2270	CAGTTGCGCAGCCTGAATGG
HP2271	TAATACGACTCACTATAGGGAACAAACGGCGGATTGAC

## Data Availability

Data sharing is not applicable for this article as no datasets were generated or analyzed during the current study.

## Supplementary Materials

The following supporting information can be downloaded at: https://www.mdpi.com/article/10.3390/microorganisms11061374/s1. Original uncropped Western and Northern blots used for creating the figures in the manuscript.
