# Supplementary material for: RNase Y Autoregulates Its Synthesis in Bacillus subtilis
Source: Microorganisms. 2023 May 24;11(6):1374. doi: 10.3390/microorganisms11061374 (PMC10303841; doi:10.3390/microorganisms11061374)

Supplementary data

Uncropped Western blots of Figure 1 C and D

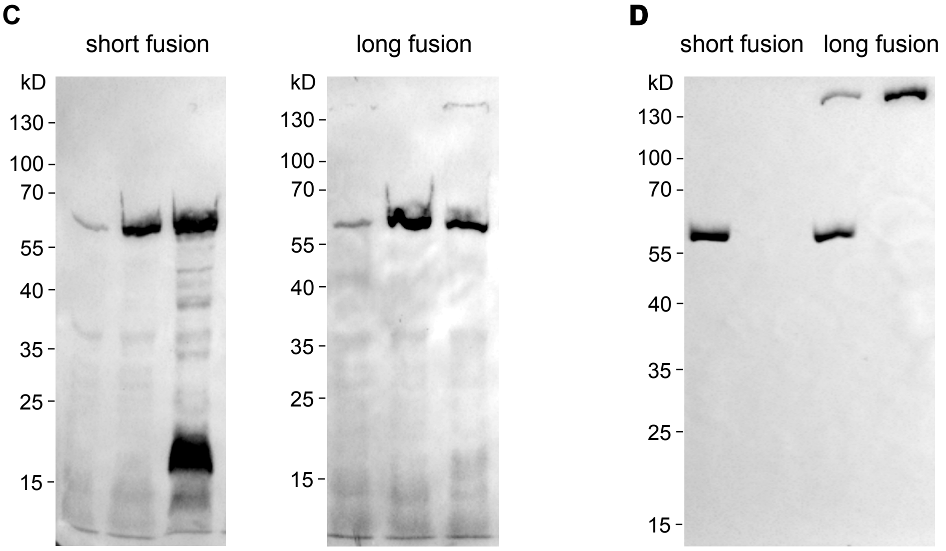

Uncropped Northern blots of Figure 2 A, B and C

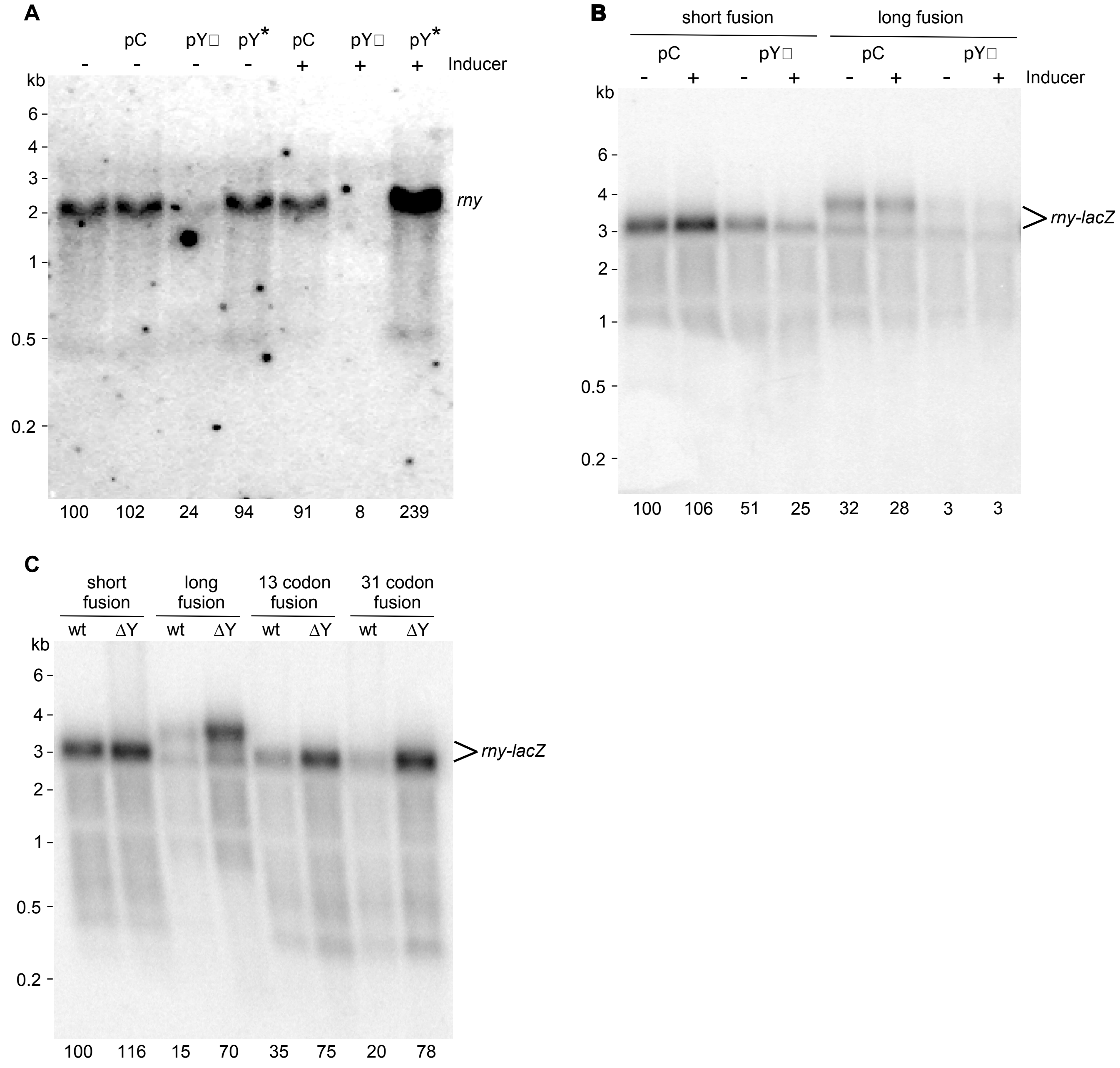

Uncropped Northern blots of Figure 3 A, B and C

A

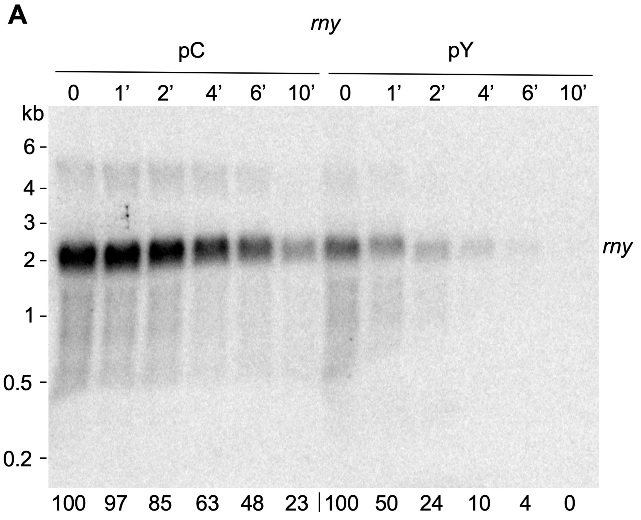

B

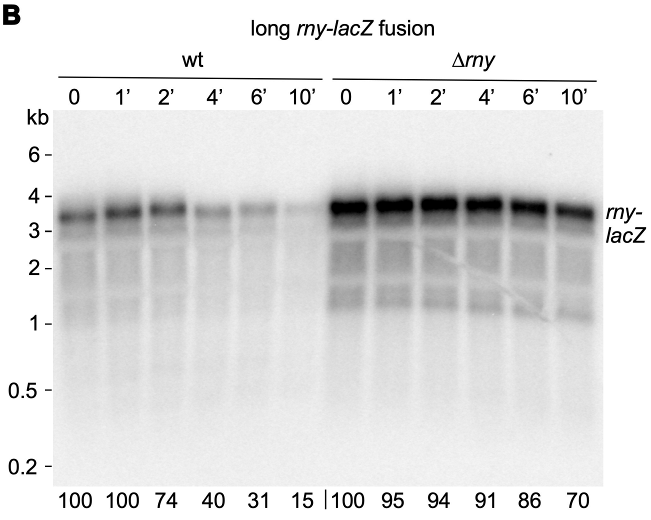

C

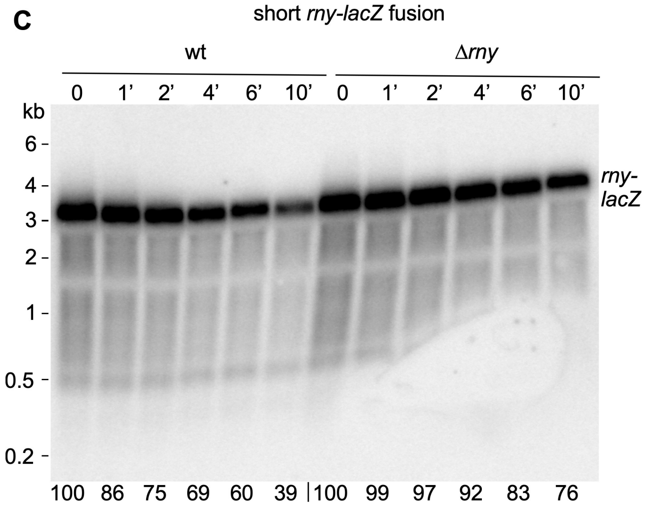

Supplement: Supplementary file 1 [file microorganisms-11-01374-s001.zip › microorganisms-2362203-supplementary.pdf]
